# Multi-tasking costs in triple-task performance despite dual-task preparation

**DOI:** 10.3758/s13421-024-01674-w

**Published:** 2025-01-28

**Authors:** Maximilian Stefani, Marian Sauter, Wolfgang Mack

**Affiliations:** 1 Department of Human Sciences, Institute of Psychology, General Psychology, University of the Bundeswehr Munich, Werner-Heisenberg-Weg 39, 85577 Neubiberg, Germany; 2 Institute of Psychology and Education, General Psychology, Ulm University, Ulm, Germany

**Keywords:** Dual task, Triple task, Task coordination, Multitasking, Training

## Abstract

This study explores multi-tasking by examining the effects of transitioning from dual-task to triple-task scenarios. Our research extends beyond conventional dual-task paradigms to investigate the impact of triple-task performance on two participant groups: those unprepared in single, dual, or triple tasks (*N* = 14) and those previously prepared in single and dual tasks (*N* = 13). The study consisted of a preparation phase with nine sessions and an assessment phase with eight sessions. In the assessment phase, both groups performed single, dual, and triple tasks of varying complexity (simple, medium, and complex). Despite the initial advantage observed in the prepared group, this advantage diminished throughout the sessions. Notably, both groups adopted distinct strategies for processing within the triple task, revealing the influence of task coordination on response times as the task set combinations expanded. The study demonstrates that preparation in the form of pre-training can facilitate applying skills acquired from specific tasks to others, with the formation of specific task pair sets playing a pivotal role in processing and coordination. Despite extensive preparation, the persistence of multi-tasking costs challenges traditional assumptions about eliminating such costs through practice. In conclusion, our research contributes to the current understanding of multi-tasking by highlighting the need for further exploration into inter(sub)task coordination and prioritization in multiple-task scenarios. The study underscores the complexities inherent in managing triple tasks and individuals' potential strategies. The findings suggest that ongoing refinement of cognitive models from dual tasks is necessary to accommodate multi-tasking behaviors in more complex environments.

## Introduction

Imagine your first driving lesson: it was likely a blend of excitement and anxiety, with every action feeling uncoordinated and overwhelming. However, as you accumulated hours behind the wheel, your ability to navigate the vehicle improved dramatically. You learned to synchronize multiple tasks – braking, shifting gears, and monitoring traffic – all aimed at achieving a goal: traveling safely from point A to point B. Fundamentally, driving involves managing several tasks simultaneously to achieve one primary objective. But what if we shift the context from driving a car to riding a motorcycle? The skills require change, and so is how these skills are coordinated.

This example highlights that real-life scenarios often require juggling more than two tasks at the same time. While research on dual tasks has given us valuable insights into how individuals handle two concurrent tasks, they may not fully capture the complexity of real-life multi-tasking situations. Understanding how people manage more than two tasks is crucial for extending cognitive theories into more ecologically valid contexts. For example, in a triple-task scenario, individuals might need to prioritize or combine tasks in new ways depending on the demands, which could reveal limitations in current models of task coordination.

Research in cognitive psychology, particularly in dual-task scenarios, shows that extended training can enhance our ability to juggle multiple tasks efficiently (Garner & Dux, 2023; Kramer et al., 1995; Liepelt et al., 2011; Schubert et al., 2017). This training leads to what is known as "transfer of learning." Transfer can be positive, where mastering one task aids in performing another, or negative, which hinders performance on subsequent tasks (for more about the taxonomy of the transfer, see Barnett & Ceci, 2002).

However, managing a goal becomes more complex as we introduce more tasks. A recent study by Stefani et al. (2022) explored this by examining behavior in triple-task scenarios, where participants had to manage three tasks simultaneously (visual-manual, visual-pedal, and auditory-vocal tasks). Unlike most dual-task studies, which typically involve only two tasks, this triple-task study found that participants grouped tasks (namely the visual-manual and visual-pedal tasks) to optimize performance. They endeavored to act correctly and efficiently while treating all tasks with equal priority. This approach to task grouping raises critical questions: Is this strategy a result of insufficient training employed to simplify the challenge of managing multiple tasks? Furthermore, can prior training in dual tasks enhance performance in triple-task scenarios? Our study builds on these inquiries, aiming to understand how training in dual-task scenarios might influence the management of more complex task combinations.

### Dual-task training

Research has consistently shown that dual-task training, where individuals practice performing two tasks simultaneously, can significantly reduce response times (RTs) and error rates (Bherer et al., 2005, 2008; Hirst et al., 1980; Kramer et al., 1995; Liepelt et al., 2011; Ruthruff et al., 2003, 2006). This training effect is not confined to specific task types but extends across various cognitive domains, enhancing the general efficiency of task performance (Strobach, 2019; Strobach & Schubert, 2017). Schumacher et al. (2001) demonstrated that with adequate training, participants could eliminate what is known as "dual-task costs" – the additional time and error typically associated with performing two tasks simultaneously compared to separately. In their study, participants engaged in concurrent visual-manual and auditory-vocal tasks, each requiring responses based on different sensory inputs. The tasks were presented simultaneously and had to be executed with equal priority. By comparing performance in dual-task to single-task conditions, Schumacher and colleagues found that participants could achieve *perfect time-sharing*, performing tasks together as efficiently as they did separately. Since the term “dual-task costs” is explicitly used to describe performance decrements in dual-task scenarios (i.e., two tasks), we propose extending this terminology by introducing “multi-task costs” as a more general definition. Multi-task costs refer to the performance decrement observed when multiple tasks are performed simultaneously compared to when performed separately. In the case of a triple task, these costs can be analyzed by comparing performance across single tasks, dual-task combinations, and the triple-task condition.

### Reasons for improvement through training

Zero dual-task costs or multi-task costs can be achieved, for example, by task automatization (Ruthruff et al., 2006; Schneider & Shiffrin, 1977), bypassing the central bottleneck (Maquestiaux et al., 2008, 2018; Ruthruff et al., 2001), by shortening the response selection (Schubert et al., 2008) or other processing stages (Dux et al., 2009; Strobach et al., 2013); see also the review by Strobach and Schubert (2017) for a detailed discussion. Notably, the major improvements were primarily observed in the auditory task, suggesting that dual-task training may preferentially enhance certain types of task processing (Schumacher et al., 2001; Strobach et al., 2012, 2013; Tombu & Jolicoeur, 2004).

One reason for such findings could be that the training may have enhanced the efficiency of response selection processes. Schubert and colleagues (2024) posit that working memory is crucial in the context of dual-task training, emphasizing that the initial setup of tasks in a dual-task scenario is fundamental to the benefits gained from such training. They propose three key hypotheses to validate the predictions of the Efficient Task Instantiation (ETI) model: firstly, dual-task coordination skills can be acquired through practice and successfully transferred to other situations; secondly, the development and execution of dual-task coordination skills are influenced by the complexity and difficulty of the individual tasks being performed simultaneously; thirdly, age-related declines in working memory capacity affect the ability to optimize and improve dual-task performance through practice (see also Schubert & Strobach, 2018; Strobach et al., 2014). However, such improvements are subject to working memory-capacity limitations (Schubert & Strobach, 2018), which involve storage, supervision, and coordination functions (Oberauer et al., 2000). As the number of tasks increases beyond two, the ability to coordinate them with the same efficiency is reduced. (Schubert & Strobach, 2018).

Another reason could be task automatization. Ruthruff et al. (2006) questioned whether the reduction of costs was possible either by integrating both tasks into one "super task" through task automatization or by shortening the processing stages. They trained participants in an auditory-verbal task, a visual-manual task, or both, and all participants were later tested in a dual-task session. They concluded that processing bottlenecks could be mitigated by automatizing at least one task, mainly when tasks are compatible and straightforward. This implies that tasks combining visual-manual and auditory-vocal elements are less likely to interfere with each other compared to other combinations (see also Göthe et al., 2016). Maquestiaux et al., (2008, 2010) provided further evidence of task automatization in younger adults, and another study emphasized the critical role of sensorimotor compatibility in task automatization (Maquestiaux et al., 2018). Strobach and Schubert (2017) outlined five conditions necessary for optimal dual-task performance and potential task automation: (1) the instruction must be to complete the task as fast as possible, (2) the compatibility of the tasks must be maintained, (3) extensive training must be provided, (4) both tasks must be given equal priority, and (5) simultaneous processing of the stimuli must be possible (e.g., same onset asynchrony of 0 ms). Despite meeting these criteria, Strobach and Schubert (2017) did not find evidence of task automation in young adults. It is important to note that their analysis was limited to dual-task trials with a specific sequence and did not encompass all possible trial permutations. This led them to conclude that post-training interference aligned more with capacity-limited processes rather than a shared capacity across tasks. However, as the ETI model indicates, improvements in RT may also be attributed to more effective task coordination.

Once participants are trained, task coordination becomes more efficient, and skills acquired in one task can often be applied to another. Liepelt et al. (2011) characterize intertask coordination (ITC) as a skill developed through extensive dual-task practice that could then be generalized across various dual-task scenarios. They showed that participants in a “hybrid” group – who trained on both single and dual tasks – did not experience significant difficulties when the tasks were varied, and consistently outperformed those who trained solely on single tasks. Consequently, they suggested that the ITC may be independent of the single tasks itself; the ITC poses additional demands. However, it is also important to note that the improvements were explicitly observed in auditory tasks, which were always executed subsequent to the visual tasks, and this applied only when one of the dual-task components was changed (see also Bherer et al., 2008). Expanding on this, Strobach et al. (2012) investigated whether skill transfer was possible when both tasks were changed, discovering that no differences emerged between groups under these conditions. Strobach et al. (2013) continued this approach by using the transfer effects to determine after which amount of time dual-task practice leads to an improvement. They concluded that enhancement likely results from reduced time required for central response selection and perceptual processing in the auditory task, providing further evidence for the ETI model (see also Strobach & Schubert, 2017).

### The previous triple-task study

In a previous study, Stefani et al. (2022) investigated participants' performance in a triple-task paradigm over three sessions, which included single, dual, and triple tasks presented in mixed blocks. Stefani et al. (2022) observed two notable findings. Firstly, RTs increased substantially in triple tasks compared to dual and single tasks. In mixed blocks, single-task RTs ranged from 689 to 971 ms, which was slower than those reported in dual-task studies with a mixed block design (see Bherer et al., 2008; Strobach & Schubert, 2017). However, direct comparisons between these studies should be made with caution due to differences in experimental designs and task complexity. Secondly, Stefani et al. (2022) observed that participants often executed the two visual tasks requiring hand and foot responses within a tight time frame (difference ≤ 100 ms), suggesting a grouping strategy to optimize task completion. Stefani et al. (2022) assumed that interference between the two visual tasks might be limited due to either (weak) crosstalk, as discussed by Koch (2009), or a bottleneck within the visual system, a concept supported within the dual-task framework by Tombu & Jolicoeur (2004) and Wickens (1981). This interference arises because both tasks compete for similar visual processing resources, leading to potential delays or reduced efficiency in task execution.

Efforts to apply the response selection bottleneck (Pashler, 1984) or the EPIC model (Meyer & Kieras, 1997a, 1997b) to predict outcomes in triple-task studies have encountered limitations. Specifically, the bottleneck model for response selection overlooks strategic or cognitive components, which are crucial for understanding complex task management. While the EPIC model incorporates strategic aspects, workload, training, and compatibility, it still falls short in providing detailed explanations for the findings of Stefani et al. (2022).

Instead, in Stefani et al. (2022), it appears that participants may have adopted a strategy of delaying their responses until both responses to the visual subtasks were prepared, resulting in nearly simultaneous execution. This raises the question of whether extensive training in triple tasks across multiple sessions would further modify or reinforce this response-grouping behavior. Additionally, it is worth investigating the potential effects of prior dual-task preparation on triple-task performance. Participants who have already mastered dual tasks may exhibit an advantage and demonstrate different performance patterns when faced with triple tasks compared to those without prior preparation.

### The current triple-task study

Initially, we established a group of participants trained in dual tasks as a *prepared group*, utilizing visual-manual and auditory-vocal tasks with zero stimulus onset asynchrony (SOA) in a mixed-block format using two-choice response alternatives. Over seven experimental sessions, all tasks remained the same. We referred to this training as the *preparation phase*. Aligned with Strobach and Schubert's (2017b) criteria for optimal dual-task performance, we anticipated that the prepared group would achieve seamless task integration, or “*perfect time-sharing,*” akin to the performance in single tasks (Schumacher et al., 2001; for a discussion about perfect time-sharing see Tombu & Jolicœur, 2004). Following the *preparation phase*, we introduced a third task during the subsequent *assessment phase*. This visual-pedal task, activated by foot pedals, was added to the established dual task, creating a triple-task scenario. The *assessment phase* was performed by both the *prepared group* from the *preparation phase* and a new *unprepared group* without prior dual-task preparation.

Based on the ETI model, even if it does not make any direct statements about differences between prepared and unprepared individuals, we expected the prepared group, who had received dual-task preparation, to perform the triple task more efficiently, with quicker responses and fewer errors than the unprepared group, who had not received any preparation (Liepelt et al., 2011; Schubert & Strobach, 2018). The preparation advantage observed in the prepared group arises from transfer effects through intertask coordination, which is independent of the component tasks trained beforehand, even when considering the addition of new tasks (Liepelt et al., 2011; Schubert & Strobach, 2018; Schubert et al., 2017; Strobach et al., 2012). Even with extensive training, we predicted that cognitive limitations prevent perfect time allocation in all three tasks. This is due to the overlapping stimulus–response (S-R) mappings in the two visual tasks (Hazeltine et al., 2006; Ruthruff et al., 2001; Sommer et al., 2001; Stefani et al., 2022) and the increased demands on task coordination and working memory (Schubert & Strobach, 2018).

## Methods

### Participants

**Fig. 1:**
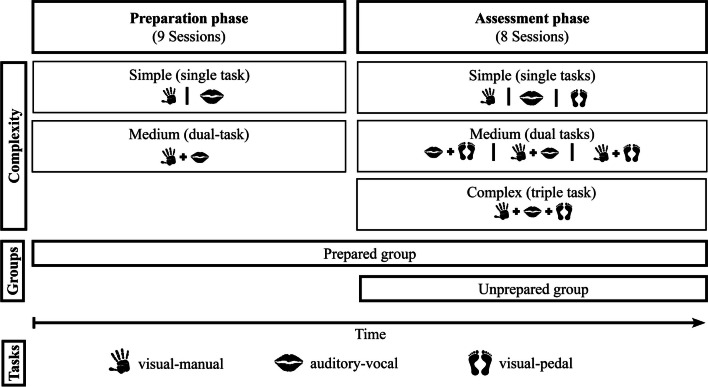
Design of the study with its phases, task complexities, and groups without the block condition. We investigated whether prepared subjects' prior knowledge of two simple and one medium task coordination complexity (preparation phase) resulted in an advantage through transfer effects. One group (prepared group) trained across nine sessions to generate prior knowledge. In the assessment phase, which now consisted of three complexities of tasks (simple, medium, and complex), another group (unprepared group) was added without prior knowledge

In the preparation and assessment phase, 13 students (four females and nine males, *M*_age_ = 22.5 years, SD = 2.8) participated as a prepared group (see Fig. 1). In the assessment phase, 14 students (five females and nine males, *M*_age_ = 22.1 years, SD = 1.7) participated as an unprepared group. Another nine participants used their right to withdraw after the preparation phase,^1^ and four from the unprepared group during the assessment phase. Conducting a power analysis with G*Power for our repeated-measures ANOVA, we aimed for a significance level (α) of 0.05 and a statistical power (1 − β) exceeding 0.9 (Faul et al., 2009). Guided by prior studies (Liepelt et al., 2011; Schubert et al., 2017; Strobach et al., 2012), we chose an effect size *F* of 0.33 considering the within-between interaction across nine sessions, each comprising three tasks (visual-manual, visual-pedal, auditory-vocal) with varying complexities (simple, medium, complex); the analysis revealed that a sample size of six participants in each group is necessary to meet these criteria. Participants attended up to nine sessions during the preparation phase and eight in the assessment phase, each lasting approximately 1 h and scheduled at least 1 day apart. Combining all the sessions completed by the two groups results in a cumulative experimentation time of 333 h. All participants had normal or corrected-to-normal vision and no hearing impairments. They provided informed consent and were given course credit or money per hour attended as compensation for their participation. All procedures performed in this study were accepted by the ethics committee of the University of the Bundeswehr Munich and in accordance with the 1964 Helsinki Declaration.

### Setup

The experiment was programmed in MatLab® 2017a with the Psychtoolbox-3.013 (Brainard et al., 2016) on a PC running Linux OS® with Ubuntu 18.04 LTS. For recruitment, we used the software ORSEE (Greiner, 2015). The study was conducted in noise-reduced medium-lit cabins. The visual stimuli were displayed on an EIZO® color monitor with a screen diagonal of 27 in. and a frame rate of 144 Hz at a resolution of 2,560 × 1,440 pixels. The BlackBox® toolkit response pad with a voice key feature was used to capture the RT of the manual and the vocal responses. The visual task was completed with the index and middle finger of the dominant hand (determined by the Edinburgh Handedness Inventory (Oldfield, 1971)) via two adjacent keys on the response pad. The Sennheiser® Model PC3 headset was used to record the voice and reproduce the auditory stimuli.

### Task

**Fig. 2:**
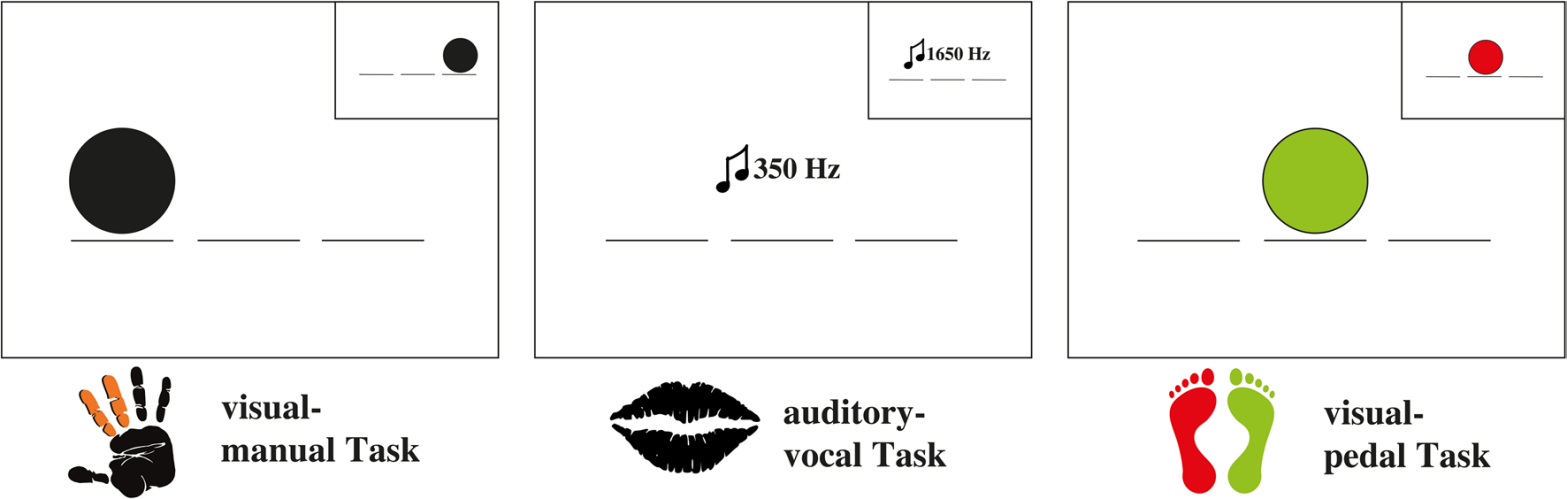
Description of the multi-tasking tasks. In the visual-manual task, the participants had to differentiate between right and left by pressing an assigned button; in the auditory-verbal task, between a high and low tone by a verbal response (high or low); and in the visual-pedal task, between green and red by pressing a button with their feet on assigned pedals

Participants performed three tasks according to three levels of complexity. Complexity is described here as coordinative complexity, defined by the amount of coordinative processing required to control and monitor the flow of information between interconnected processing steps (Mayr & Kliegl, 1993). We distinguish between simple (the three single-tasks), medium (the three dual-tasks), and complex (the triple-task) tasks, whereas all single tasks (or all sub-tasks in the dual and triple tasks) were based on two-choice RT alternatives. The tasks were a visual-manual task, an auditory-vocal task task, and a visual-pedal task (see Fig. 2). Each task was generally structured as follows: first, participants were presented with a fixation screen for 500, 1,000, or 1,500 ms with a fixation cross in the center (1° diameter) of the screen and in the preparation phase, two horizontal dashes and in the assessment phase, three horizontal dashes (5° width 5 pixels high) positioned slightly below the centers of the circles as a placeholder. There was a change in the fixation screen, as the assessment phase now included three possible circle positions, one of which was in the center where the fixation cross was previously displayed. The stimulus remained for 100 ms, and the trial ended when the participant responded. After the response, the trial ended with the presentation of the RT for 700 ms.

In the visual-manual task, participants responded manually to a white circle (2.5° radius) appearing randomly and equally distributed at the left or right position (each at 9.0° distance from the middle) arranged horizontally on the screen by pressing a button (left position with the index finger and right position with the middle finger). In the auditory-vocal task, participants responded vocally via the voice key when hearing a sine wave tone at frequencies of either 350 or 1,650 Hz by saying "LOW" or "HIGH" (German: "TIEF" or "HOCH"). In the visual-pedal task, participants responded with foot pedals discriminating between red (RGB: 255,0,0, left foot) and green (RGB: 0,0,255, right foot), which colored the white circle.

A single-task, dual-task, or triple-task could be presented as a function of session and block condition. Thus, participants performed pure single-task blocks, mixed dual-task blocks (consisting of single and dual tasks), and mixed triple-task blocks (consisting of single, dual, and triple tasks). Single tasks had a simple complexity level, dual tasks had a medium complexity level, and the triple-task complexity level was complex. The dual-task could be a visual-manual—auditory-vocal, visual-manual—visual-pedal, or visual-pedal—auditory-vocal task. The triple-task consisted of all three tasks.

In the dual tasks and the triple task, participants had to respond to all two or three tasks as fast as possible (tasks presented with an SOA of 0 ms).

### Design

The experiment was structured in two main phases. Initially, a subset of participants underwent preparation (training) during the *preparation phase*, where they were exposed to simple and medium complexity tasks, thereby achieving the status of prepared participants. Subsequently, in the *assessment phase*, both the prepared participants (from the preparation phase) and unprepared participants (who had no prior exposure to the tasks) received training.

#### Preparation phase

In each session, participants had to perform three different types of experimental blocks. Two blocks were pure single-task blocks, respectively, with only visual-manual or auditory-vocal single-tasks, and the third block was a mixed dual-task block with both single-tasks and dual-tasks. Each session always started with two pure single-task blocks, alternating two mixed dual-task blocks and one of the two pure single-task blocks. In a pre-session, each participant passed through each of the three possible blocks to become familiar with the tasks (the pre-session was not included in the results). In session 1, eight mixed dual-task blocks and six pure single-task blocks were completed. Two additional mixed dual-task blocks had to be performed from the second session onwards. Each pure single-task block consisted of 24 single-task trials, and each mixed dual-task block consisted of 12 auditory-vocal single-tasks, 12 visual-manual single-tasks, and 28 visual-manual – auditory-vocal dual-tasks.

#### Assessment phase

In eight training sessions, participants had to perform five different types of experimental blocks. The first three experimental blocks comprised three pure single-task blocks with only one of the three single tasks each, the fourth experimental block comprised one mixed dual-task block with all three single-tasks and three dual-task combinations, and the fifth experimental block comprised one mixed triple-task block with all single tasks, dual tasks, and the triple task. In each session, three single-task blocks were followed by two mixed dual-task blocks, two mixed triple-task blocks, one mixed dual-task block, and one mixed triple-task block. Each single-task block consisted of 12 single-task trials, each dual-task mixed block consisted of eight tasks for every single-task and 12 tasks for each dual-task, and each triple-task block had, in addition to the tasks from the dual-task block, 24 triple tasks.

Participants were instructed to perform all the tasks as quickly and accurately as possible. They were told not to respond in a particular order and to prioritize all tasks equally.

#### Analyses

Verbal responses were analyzed when the voice key was triggered using the R-Package "VoiceExperiment" (Nett, 2017). For uncertain audio analysis results, trials were listened to and classified manually. RStudio 2023.06.1 (RStudio Team, 2016) and R 4.3.1 were used with the tidyverse package version 1.3.0 (Wickham, 2017) for data preparation and creating figures and JASP version 0.17.3 to calculate repeated-measures ANOVAs and post hoc analyses. We calculated mean RTs and error rates per participant and condition if not stated otherwise. Post hoc tests were conducted on the estimated marginal means with Bonferroni's *t*-test, and we used the Bonferroni equation for computing degrees of freedom.

### Transparency and openness

We report how we determined our sample size, all data exclusions (if any), all manipulations, and all measures in the study. All data, analysis code, and research materials are available via the Open Science Framework at https://osf.io/fr4wa/. This study's design and its analysis were not pre-registered.

## Results

### Inclusion and exclusion of data

Trials with RTs below 80 ms and above 4,500 ms were removed (*preparation phase*: simple tasks: 1.2% and medium tasks: 1.0%; *assessment phase:* simple tasks: 1.1%, medium tasks: 0.9%, and complex tasks: 1.0%). These RTs came about when participants spoke too softly (RT > 4,500 ms) or made noises during the task (RT < 80 ms). Only correct trials were included in the RT analyses. This means that all (sub)tasks had to be executed correctly.

### Preparation phase

The preparation phase served to train participants on performing the visual-manual and auditory-vocal tasks concurrently as a dual task. Since the preparation process itself was not the primary focus, details related to the preparation are provided in the Appendix Tables 2 and 3. This section analyzes only the final session 9 of the preparation phase to establish the baseline performance level of the prepared participants before proceeding to the assessment phase.

#### RT: Last session

**Fig. 3:**
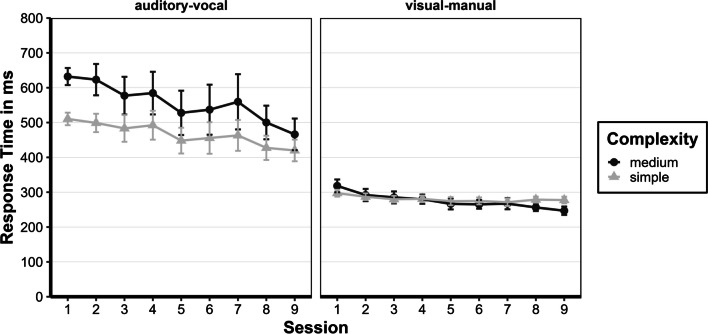
Response times for all sessions of the preparation phase. Mean response times of the preparation phase of the prepared group are given in ms (calculated from individual participant's means) as a function of complexity (simple or medium), task (auditory-vocal or visual-manual), and session (1–9). Error bars represent the ± 1 standard error of the mean

To describe their status after nine sessions of preparation, a repeated-measures ANOVA with Greenhouse–Geisser corrections for violations of sphericity was performed with the within-subject factors complexity (simple and medium) and task (visual-manual and auditive-vocal) for session 9. The main effect of complexity was not significant, *F*(1, 12) = 0.53, *p* = 0.48, *η*^*2*^*p* = 0.042. The main effect of the task was significant, *F*(1, 12) = 37.9, *p* < 0.001, *η*^*2*^*p* = 0.759. Participants responded faster to the visual-manual task (*M* = 262 ms) than to the auditive-vocal task (*M* = 443 ms). The interaction between task and complexity was significant, *F*(1, 12) = 17.48, *p* = 0.001, *η*^*2*^*p* = 0.593 (see Fig. 3). The complexity level of the visual-manual task did not differ (*M* = −31 ms, 95% CI [−71; 10], *p*_*bonf*_ = 0.229), but the complexity level of the auditive-vocal task did (*M* = 46 ms, 95% CI [6; 86], *p*_*bonf*_ = 0.019).

#### Error rate (ER): Last session

**Fig. 4:**
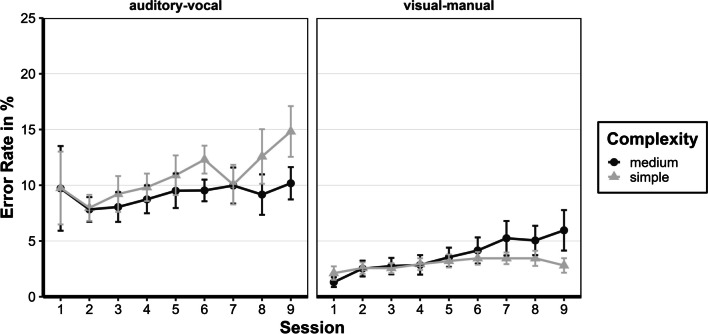
Error rates for all sessions of the preparation phase. Mean error rates of the preparation phase of the prepared group are given in percentage (%) (calculated from individual participant's means) as a function of complexity (simple or medium), task (auditory-vocal or visual-manual), and session (1–9). Error bars represent the ± 1 standard error of the mean

To assess participants' status after nine preparation sessions, a repeated-measures ANOVA with Greenhouse–Geisser corrections for violations of sphericity was used, similar to the RTs. The main effects for complexity, *F*(1, 12) = 9.7, *p* = 0.009, *η*^*2*^*p* = 0.447, and task, *F*(1, 12) = 34.95, *p* < 0.001, *η*^*2*^*p* = 0.744, as well as the interaction between complexity and task, *F*(1, 12) = 25.33, *p* < 0.001, *η*^*2*^*p* = 0.679, were significant. The complexity simple and medium were significantly different (*M* = 4.6%, 95% CI [1.4; 7.8], *p*_*bonf*_ = 0.009), and participants made fewer errors in the visual-manual task than the auditory-vocal task (*M* = 6.5%, 95% CI [4.1; 8.9], *p*_*bonf*_ < 0.001), but this difference was only observed in the complexity simple (*M* = 12.5%, 95% CI [7.8; 17.2], *p*_*bonf*_ < 0.001) (see Fig. 4).

### Assessment phase

The analysis aims to address three questions. First, does the group that underwent dual-task preparation exhibit a performance advantage over the unprepared group, especially in the first session of the assessment phase? Second, do participants tend to group the two visual tasks together in the last session after training, mirroring the pattern observed in a preceding study involving a triple-task scenario? Third, are multi-task costs persistent in the last session of the assessment phase? Despite the anticipated improvements from dual-task preparation, it is important to identify and quantify any residual challenges participants face when managing multiple tasks. An analysis of the training process can be found in the Appendix Tables 4 and 5.

#### RT: First session

**Fig. 5:**
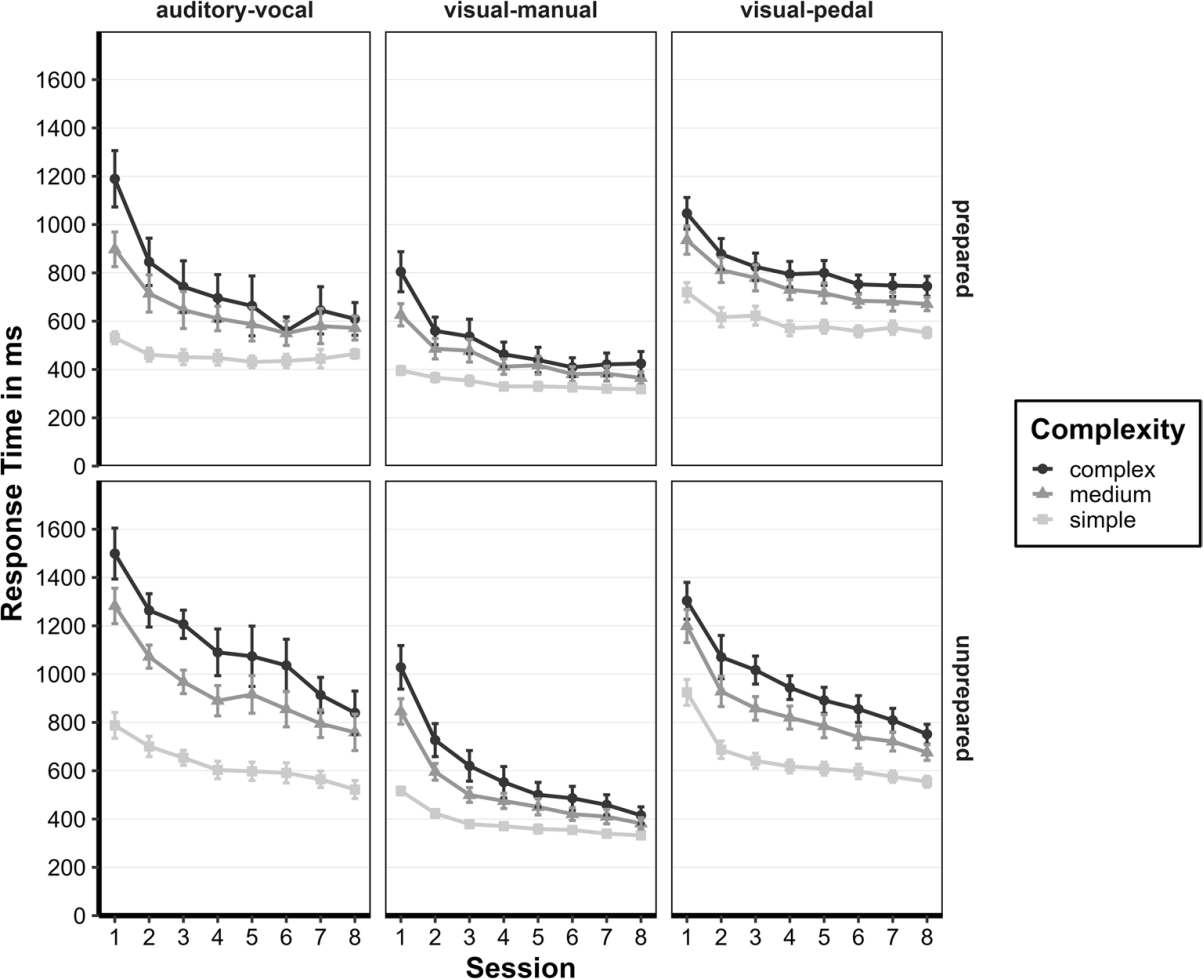
Response times for all sessions of the assessment phase. Mean response times of the assessment phase of the prepared and unprepared group are given in ms (calculated from individual participant's means) as a function of complexity (simple, medium, or complex), task (auditory-vocal, visual-manual, visual-pedal), and session (1–8). Error bars represent the ± 1 standard error of the mean

We calculated a repeated-measures ANOVA with Greenhouse–Geisser corrections for violations of sphericity with the within-subject factors complexity (simple, medium, and complex) and task (visual-manual, visual-pedal, and auditory-vocal) and between-subject factor preparation status (prepared and unprepared) for session 1 (see also Fig. 5). The main effect for the preparation status was significant, *F*(1, 25) = 9.98, *p* = 0.004, *η*^*2*^*p* = 0.285. The prepared group (*M* = 805 ms) responded *M* = 239 ms, 95% CI [84; 396] faster than the unprepared group (*M* = 1,045 ms). Participants needed for the complexity complex (*M* = 1,150 ms) more time than for medium (*M* = 948 ms) and for medium more time than for simple (*M* = 691 ms), *F*(1.21, 30.27) = 156.60, *p* < 0.001, *η*^*2*^*p*​ = 0.862, and was fastest in the visual-manual task (*M* = 702 ms), with no differences between the visual-pedal (*M* = 1,042 ms) and auditory-vocal task (*M* = 1,045 ms), *F*(1.84, 45.90) = 49.26, *p* < 0.001, *η*^*2*^*p*​ = 0.663. The interaction between complexity and task was also significant, *F*(2.34, 58.61) = 20.19, *p* < 0.001, *η*^*2*^*p* = 0.447, whereas participants responded in complexity simple with visual-manual (*M* = 486 ms) → auditory-vocal (*M* = 685 ms) → visual-pedal (*M* = 889 ms) but differently in medium visual-manual (*M* = 689 ms) → visual-pedal (*M* = 1,048 ms) auditory-vocal (*M* = 1,093 ms) and complex visual-manual (*M* = 916 ms) → visual-pedal (*M* = 1,175 ms) → auditory-vocal (*M* = 1,344 ms). The interaction between task and preparation status, *F*(1.84, 45.90) = 1.90, *p* = 0.165, *η*^*2*^*p*​ = 0.071, and complexity, task, and preparation status was not significant, *F*(2.34, 58.61) = 0.51, *p* = 0.631, *η*^*2*^*p*​ = 0.020.

#### ER: First session

**Fig. 6:**
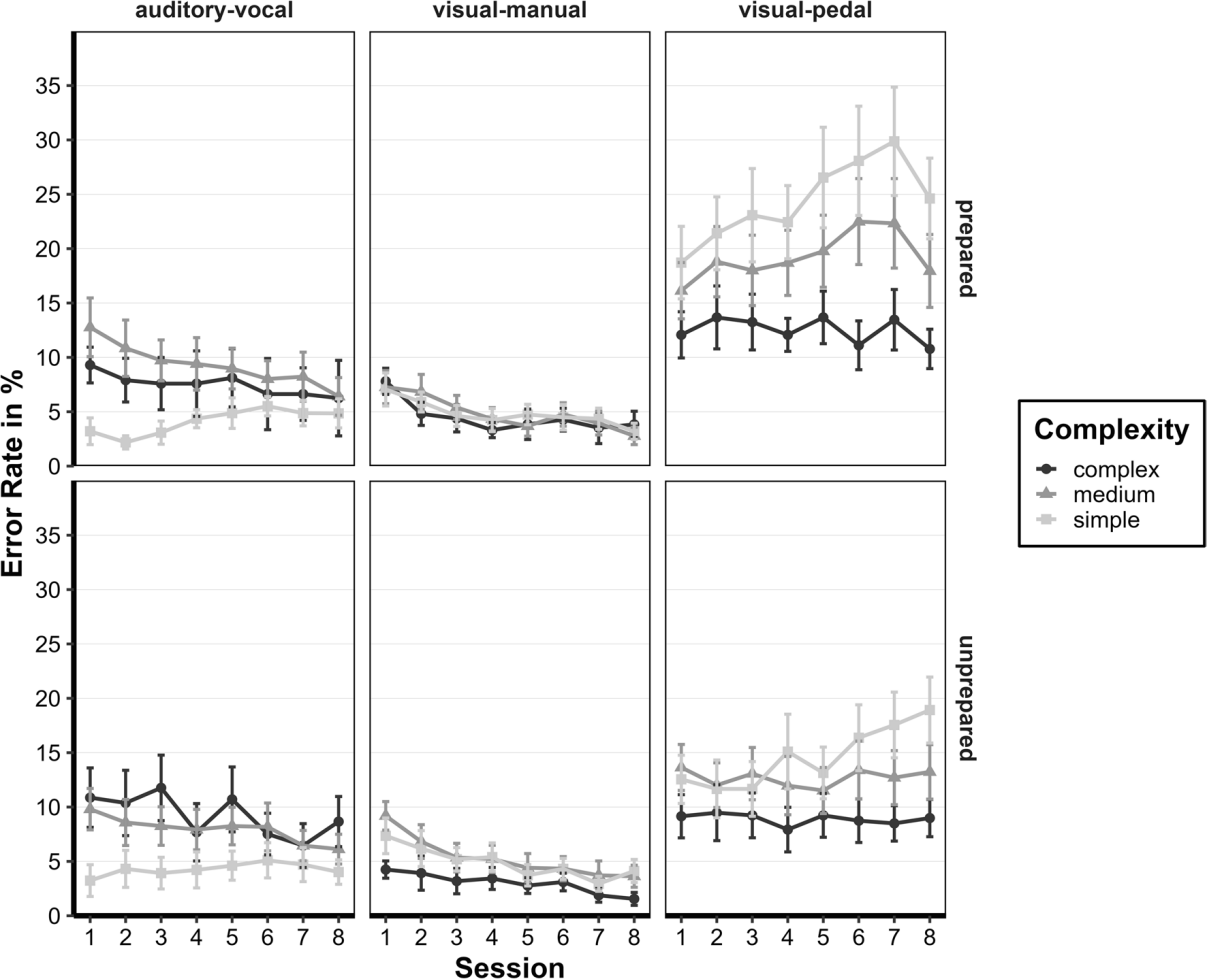
Error rates for all sessions of the assessment phase. Error rates of the assessment phase of the prepared and unprepared group are given in ms (calculated from individual participant's means) as a function of complexity (simple, medium, or complex), task (auditory-vocal, visual-manual, visual-pedal), and session (1–8). Error bars represent the ± 1 standard error of the mean

We conducted the same repeated-measures ANOVA with Greenhouse–Geisser corrections for violations of sphericity for the error rate, similar to the RTs (see Fig. 6). The preparation status's main effect was insignificant, F(1, 28) = 0.63, p = 0.433, η2p < 0.001. The main effects for complexity, F(1.98, 55.50) = 11.03, *p* < 0.001, η2p < 0.001, and for task, F(1.46, 40.96) = 14.51, *p* < 0.001, η2p < 0.001, were significant. The interaction between complexity and task was also significant, F(3.08, 86.36) = 15.60, *p* < 0.001, η2p < 0.001, whereas participants made different errors dependent on complexity; simple: auditory-vocal (M = 3.1%) → visual-manual (M = 7.1%) → visual-pedal (M = 15.5%) but differently in medium: visual-manual (M = 8.122%) → auditory-vocal (M = 11.180%) → visual-pedal (M = 14.782%) and complex: visual-manual (M = 5.918%) → auditory-vocal (M = 9.975%) → visual-pedal (M = 10.506%). There was no interaction between task and preparation status, F(1.46, 40.96) = 1.13, p = 0.316, η2p < 0.001. There was a significant interaction with complexity, task, and preparation status, F(3.08, 86.36) = 3.23, p = 0.025, η2p < 0.001. The interaction effect is based on the differences in the auditory-vocal task, with the prepared group producing the most errors in the complexity medium (M = 12.8%) and the unprepared group in the complexity complex (M = 10.9%).

#### RT: Last session

**Fig. 7:**
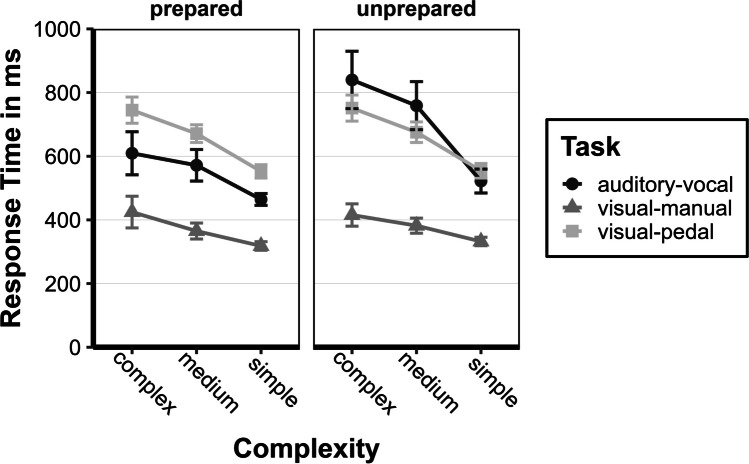
Response times of the prepared and unprepared group in the last session of the assessment phase. Mean response times for the last session (last session of the assessment phase) are given in ms (calculated from individual participant's means) for the prepared and unprepared group as a function of complexity (simple, medium, or complex) and task (auditory-vocal, visual-manual, or visual-pedal). Error bars represent the ± 1 standard error of the mean

For the last session in the *assessment phase*, we calculated a repeated-measures ANOVA with Greenhouse–Geisser corrections for violations of sphericity with the within-subject factors complexity (simple, medium, and complex) and task (visual-manual, visual-pedal, and auditory-vocal) and between-subject factor preparation status (unprepared and prepared) for the last session (see also Fig. 7). We observed no significant differences between the unprepared and prepared groups *F*(1, 25) = 1.31, *p* = 0.262, η2*p* = 0.050, but descriptively a difference of 55 ms, 95% CI [−153; 44] could still be observed (*M*_*prepared*_ = 535 ms, *M*_*unprepared*_ = 590 ms). However, the analysis of the individual tasks across all sessions revealed a significant difference in the main effect for the auditory-vocal task (see Table 4 in the Appendix. Furthermore, independent of the preparation status (interaction between complexity and preparation status, *F*(1.10, 27.56) = 0.97, *p* = 0.341, η2*p* = 0.038), both groups required more time to perform the tasks with increasing complexity (main effect for complexity, *F*(1.10, 27.56) = 38.02, *p* < 0.001, η2*p* = 0.603). Post hoc tests showed significant differences between all levels of complexity (*ps* < 0.002): complex (*M* = 631 ms) > medium (*M* = 571 ms) > simple (*M* = 487 ms). Furthermore, visual-manual (*M* = 381 ms) was generally performed faster than visual-pedal (*M* = 681 ms) or auditory-vocal (*M* = 626 ms). However, there was no difference between visual-pedal and auditory-vocal (the main effect of the task, *F*(1.49, 37.25) = 69.48, *p* < 0.001, η2*p* = 0.735). The interaction of task and preparation status, *F*(1.49, 37.25) = 5.68, *p* = 0.012, η2*p* = 0.185, further showed that prepared participants favored visual-manual (*M* = 376 ms) to auditory-vocal (*M* = 546 ms) and auditory-vocal to visual-pedal (*M* = 684 ms) and the unprepared visual-manual (*M* = 386 ms) to visual-pedal (*M* = 678 ms) to auditory-vocal (*M* = 706 ms) in order to process the tasks. However, auditory-vocal and visual-pedal RTs were not significantly different within the two groups. It did indicate, however, that prepared participants preferred the previously trained tasks (visual-manual and auditory-vocal). The difference in RTs of responding to visual-pedal and auditory-vocal differed between the two groups, which was evident in the interaction effects of complexity and task, *F*(1.97, 49.18) = 16.23, *p* < 0.001, η2*p* = 0.394, as well as in complexity, task, and preparation status, *F*(1.97, 49.18) = 4.12, *p* = 0.023, η2*p* = 0.141. Across all complexities, there was no significant difference in any of the groups between visual-pedal and auditory-vocal, except in the prepared group when the tasks were simple, *M* = 172 ms, 95% CI [43; 335]. Descriptively, however, a difference of *M* = −135 ms, 95% CI [−299; 28] could be observed in the complexity complex between auditory-vocal and visual-pedal in the prepared group and *M* = 88 ms, 95% CI [−69; 246] in the unprepared group. For a better overview of the differences between the subtasks (inter-response intervals) in the complexity complex, see Table 1.

**Table 1:** Inter-response interval in the last session of the assessment phase

Task 1 – Task 2	Prepared		Unprepared	
	*M*	*95% CI*	*M*	*95% CI*
Visual-manual **–** auditory-vocal	185	[21; 348]	424	[267; 582]
Visual-manual **–** visual-pedal	320	[157; 484]	336	[178; 494]
Visual-pedal **–** auditory-vocal	135	[−28; 299]	88	[216; 559]

Mean differences of response times of the complex sub-tasks in ms for the last session (calculated from individual participant's means) for the prepared and unprepared group. Values were constantly reported to be positive

#### ER: Last session

We calculated the same repeated-measures ANOVA with Greenhouse–Geisser corrections for violations of sphericity for the error rate for the last session. We observed no significant differences between the two groups, *F*(1,25) = 0.302, *p* = 0.587, η2*p* = 0.012. Furthermore, no interaction with the factor preparation status was significant, complexity and preparation status, *F*(1.65, 41.15) = 0.11, *p* = 0.861, η2*p* = 0.004; task and preparation status, *F*(1.37, 34.22) = 0.81, *p* = 0.411, η2*p* = 0.031; complexity, task and preparation status, *F*(2.84, 71.05) = 1.25, *p* = 0.297, η2*p* = 0.048. The main effects for complexity, *F*(1.65, 41.15) = 9.20, *p* = 0.001, η2*p* = 0.269, and task, *F*(1.37, 34.22) = 33.12, *p* < 0.001, η2*p* = 0.570, were significant as well as the interaction between complexity and task, *F*(2.84, 71.05) = 15.41, *p* < 0.001, η2*p* = 0.381. Most errors occurred in the simple tasks (10.1%), followed by medium (8.6%) and complex tasks (6.5%). In the visual-pedal task, the most errors were observed across all complexities (16.0%), followed by the auditory-vocal task (6.0%) and visual-manual task (3.2%). Interestingly, participants made significantly fewer errors in the visual-pedal task with rising complexity (21.9% → 16.2% → 10.0%; *ps* < 0.001), unlike the other two tasks, where the error rates remained statistically constant across the complexity.

## Discussion

The majority of multi-tasking research is based on dual-task paradigms. Nevertheless, we argue that refining the current models requires extensions to scenarios with more than two concurrent tasks. The present study builds on the premise that triple-task performance is systematically distinct from dual-task performance (Stefani et al., 2022). We examined the impact of a triple-task scenario on two participant groups: one with no prior preparation and another who had previously prepared (trained) on two of the three tasks involved in the form of single and dual tasks.

### Assessment of preparation benefits

We initially hypothesized that participants could gain an advantage by preparation (training) on part of the upcoming triple task beforehand. The expectation was that this preparation would enhance performance on the familiar tasks and the subsequent unfamiliar tasks. While the first seems logical at first glance, it is not immediately logical on closer inspection. The two tasks were precisely the same, but in the *assessment phase*, they occurred in a different setting, i.e., the possible response combinations increased rapidly with the third task (triple-task mixed blocks consisted of the three single tasks, three dual tasks, and the triple task). During the preparation phase, participants had to manage two possible task sets with three possible combinations: two single tasks and one dual task. Introducing the third task more than doubled the task set combinations to seven. One group underwent preparation with both the visual-manual and auditory-vocal tasks to evaluate the impact of preparation. This preparation led to notable improvements in the visual-manual task, where participants managed to perform without incurring additional time costs (*M* = −31 ms). In contrast, the auditory-vocal task continued to present a response delay (*M* = 46 ms). This means that contrary to other training studies, statistically interference could only be reduced in the visual-manual task at the end of session 7 in the *preparation phase*. For the auditory-vocal task (complexity medium vs. simple), a difference of 46 ms could still be observed. This partial improvement aligns with findings from Liepelt et al. (2011), where participants also showed enhancements in only one of the dual tasks. However, the results of Lyphout-Spitz et al. (2024) showed that the central bottleneck could be bypassed even if costs can still be observed. Lyphout-Spitz et al. found evidence contradicting prevailing theories: participants could perform two novel tasks in parallel without a central bottleneck, if they are properly prepared – especially by boosting preparation for the second task through intermixing single-task trials. Nearly half the participants were able to bypass the central bottleneck when preparation for the second task was boosted in this way.

Furthermore, compared with Schumacher et al. (2001) and Liepelt et al. (2011), we observed significantly higher error rates. The error rates in the dual tasks (complexity medium) were almost twice as high as in Schumacher et al. (2001) and Liepelt et al. (2011). However, the methodological structure differs in two essential factors: it was only necessary to differentiate between two and not three possible answers in the present experiment, and the participants received no reward (compensation in any way) for each correct answer. Possibly, first, the combination of fewer choices; second, the instruction to be as fast as possible (but also to answer as correctly as possible); and third, the RT feedback after each trial led to an increased number of wrong answers, which may be the result of trading off speed and accuracy in favor of speed. However, only the preparation status was relevant to our further research question. While the *preparation phase* offered some expected benefits, the complexities introduced by adding a third task reveal a deeper layer of interaction between task performance and cognitive coordination.

### Impact of task complexity on coordination and performance

The intricacies of task coordination become even more pronounced as we consider the role of task complexity on performance, where subtle differences in coordination strategies emerge. In particular, both groups coordinated the three subtasks within the triple task slightly differently (which could only be observed descriptively in the inter-response intervals; see Table 1). Previous observations by Stefani et al. (2022) indicated that participants tended to synchronize their responses to both visual tasks, responding to them in a closely matched timeframe. However, these findings were based on shorter training durations where random variation could have influenced the results. In contrast, our study, which involved more extensive training, did not confirm this pattern of synchronized responses. Instead, we found that the trained participants' response patterns varied depending on their prior preparation experience.

We had anticipated that the most effective coordination strategy, or intertask coordination (ITC), would involve standard pairings of modality-specific features within the triple task, as these pairings could be processed more efficiently and rapidly, as Göthe et al. (2016) suggested. Nonetheless, the actual behavior of participants diverged from this expectation. Those with dual-task preparation tended to complete the familiar tasks first before addressing the third, newly introduced task. Consequently, the trained and untrained groups developed distinct ITC strategies based on their experience. Moreover, the variety of task set combinations provided appeared to impact ITC. We noted performance variances across tasks of differing complexity, and the simple tasks elicited slower RTs than those reported in dual-task research. This disparity could be attributed to our study's broader range of task combinations.

De Jong (1995) and Gottsdanker (1979) have already investigated the difference between homogeneous (only one task set occurs) and mixed blocks (multiple task sets). Both found significant differences between the two blocks. Participants prepared for a particular task and then responded faster to that task than when another task was required. However, Tombu and Jolicœur (2004) doubted whether the effect could be generalized, as Schumacher et al., (2001; see also Strobach et al., 2013) did not observe any differences between single tasks in homogeneous and mixed blocks. Despite this, similar to De Jong (1995) and Gottsdanker (1979), we observed significant differences in descriptives compared to mixed blocks with these studies. In homogeneous blocks, participants can focus on a single task set, which is the mental configuration required to execute a specific task, including relevant sensory information, responses, and cognitive processes. In contrast, mixed blocks involve multiple task-set combinations, requiring participants to switch between them. This switching process incurs a "switch cost" regarding increased RT and decreased accuracy due to the need for additional cognitive processes to reconfigure the mental state for the next task (Mayr & Kliegl, 2000). According to Mayr and Kliegl (2000), task sets can be viewed as intricate retrieval structures, with multiple task sets interconnected through a shared "experimental context" node. The process of alternating between two or more tasks can be visualized as shifting the focus of working memory along these retrieval paths. The challenge of internal control lies in correctly positioning the focus of one's working memory within the pertinent retrieval structure. In mixed blocks, participants cannot focus on just one task; thus, they stay in the "experimental context" to reduce the average retrieval distance to all possible task sets. Our study involved a triple-task paradigm with three possible task sets, an increase from the typical dual-task studies. This increase in task sets and task-set combinations leads to greater uncertainty and coordinative complexity, resulting in longer RTs. The probability of preparing correctly decreased significantly from 1:3 in dual-task studies to 1:7 task-set combinations in our triple-task study. In real-world scenarios, such as driving a car, the probabilities may be much lower, as dozens of potential tasks must be coordinated depending on the traffic situation, which requires managing and switching between multiple task sets.

In exploring how participants manage multiple tasks, we noted a pronounced preference for the visual-manual task. This aligns with findings from Schumacher et al. (2001), where similar tendencies were reported. Recognizing these task preferences is pivotal as it provides a lens through which we can interpret multi-tasking costs that arise with escalating task complexity. These costs were evident in the performance of both prepared and unprepared participants, underscoring the cognitive load it imposes.

### Alternative explanation for different inter-response intervals

Hirsch et al. (2021) propose that participants develop a hierarchical control system during dual-task preparation that stores the individual tasks and the entire task-pair set. This suggests that when faced with a dual task, participants retrieve the combination of the two tasks rather than the individual tasks themselves. Applying this concept to our study, it is plausible that the dual-task preparation in the preparation phase created a task-pair set that is now being retrieved, even with the addition of a third task in the assessment phase. Instead of creating an entirely new set for the triple task, the existing (learned) task-pair set from the dual-task preparation may be extended to incorporate the additional task. In the case of the unprepared participants, who are facing all tasks as new, a task-pair set is likely formed in a manner similar to what has been observed in dual-task research. Specifically, the auditory-vocal task tends to be processed last after completing the other tasks. These insights suggest that the formation and retrieval of task sets play a crucial role in how participants coordinate and execute multiple tasks, with prior preparation influencing the strategies employed when faced with novel task combinations.

### Interpretation of multi-task costs

Multi-task costs are a central aspect of our study as they provide a measure of the cognitive load and efficiency in task execution. Traditionally, these costs have been evaluated by comparing performance on dual tasks to single tasks, as detailed in seminal works by Schumacher et al. (2001) and Tombu and Jolicœur (2004). The assumption is that with sufficient training, participants can improve their allocation of cognitive resources, leading to enhanced response selection and potential task automatization. Ideally, with extensive practice, multi-task RTs could approach those of single tasks, potentially reducing multi-task costs to zero.

However, our study presents a more complex scenario. Despite the training, differences in RTs and error rates persisted across all levels of task complexity throughout the training sessions, indicating enduring multi-task costs. This suggests that the number of tasks and the coordination required to manage them are substantial in these costs. While classic dual tasks increase coordination demands by the number of possible responses, our study introduces the additional complexity of multiple stimuli and their associated response combinations. It appears that this coordination could be so working memory demanding that either the duration of our training was insufficient, or it may be inherently challenging to eliminate multi-task costs entirely (see also, Schubert et al., 2024). Therefore, the effective coordination of multiple subtasks appears to be more critical when the task combinations and task sets expand and should be a focal point in cognitive modeling.

### Triple tasks in the context of multi-tasking models

Our findings validate the impact of training on task performance, illustrating distinct improvements across various task conditions. It is theorized that such training develops specialized task sets, enhancing the efficiency of these tasks (Schubert & Strobach, 2018). This enhancement in executive functions, particularly regarding coordination and resource allocation (cf. Strobach et al., 2014), equips participants with skills that lead to a more effective activation of these task sets in working memory. We anticipated this would give prepared participants an edge in the triple tasks, an advantage evident in the earlier stages. Although prepared participants initially showed quicker RTs across all task complexities despite introducing a new task, by the end of the assessment phase, the performance gap between the prepared and unprepared groups had almost closed (55-ms difference), yet significant multi-task costs persisted.

The frameworks for multi-tasking, such as EPIC (Meyer & Kieras, 1999) and Wickens' resource model (Wickens, 1981), seek to predict task-related processes and behaviors. These models have been instrumental in understanding single and dual tasks within laboratory settings but face challenges when applied to more complex, real-world multi-tasking scenarios. Klein et al. (2003) distinguish between the micro-cognitive analysis typical of lab studies and the macro-cognitive analysis necessary for real-world applications, noting the limited ecological validity of the former. Indeed, Hoffmann et al. (2019) have highlighted the inadequacies of these models in accounting for the strategies and task prioritizations that emerge from training, which are not adequately explained by the structural predictions of EPIC or the resource allocations proposed by Wickens.

In particular, structural models like EPIC assume that tasks can be processed with equal speed and efficiency, whether in isolation or combination. However, training studies reveal that this assumption holds for only a subset of tasks, often just one within a more complex array (Pashler, 1994). This suggests that the efficiency of task processing in dual-task situations can be influenced by various factors, including the nature of the tasks, the individual's experience with the tasks, and the specific demands of the tasks. As demanded by triple tasks, the physical coordination required for multiple simultaneous actions introduces a level of coordinative complexity that can exceed our capacity for synchronicity. This leads to a shift from coordinative to sequential complexity (Mayr & Kliegl, 2000), much like the linguistic requirement to articulate words in sequence to form coherent sentences. In such cases, the central bottleneck is not necessarily cognitive but rather stems from the biomechanical constraints of our effectors.

The EPIC model also does not account for individuals' varied prioritizations or strategies, demonstrated even in dual-task conditions (Hommel, 2020). Similarly, while insightful, Wickens' resource model does not fully encapsulate the dynamic prioritization that can result from the interplay between multiple tasks and the available cognitive resources.

More recent approaches, such as Strobach's dual-task coordination adjustment framework (2024), offer a fresh perspective on how coordination demands are managed during multi-tasking. Although this framework is primarily focused on dual-task situations, its core ideas may also shed light on triple-task conditions. Strobach emphasizes the importance of adaptive adjustments in coordination strategies, which depend on both task requirements and individual experience. In the context of our findings, it seems likely that participants employed similar adaptive strategies to handle the demands of triple tasks during training. However, the persistent multi-task costs observed in our results suggest that these adaptive mechanisms may reach their limits when moving from dual- to triple-task scenarios, where the complexity of coordination increases significantly.

Given these considerations, our study suggests that existing multi-tasking models may require re-evaluation and expansion to incorporate the complexities observed in training effects, particularly in scenarios that involve more than two tasks. This highlights the necessity for further research to probe deeper into the nature of triple-task scenarios and refine the predictive power of multi-tasking frameworks for complex, real-life environments.

### Limitations of the study

Our investigation into multi-tasking has yielded insights that contribute to understanding this complex cognitive process. However, recognizing the limitations of our study is essential for accurately interpreting and applying our findings. Primarily, the task design aimed to emulate real-world multi-tasking complexity may not fully capture the unpredictable nature of such scenarios outside the laboratory. The controlled lab environment, while necessary for experimental rigor, might not reflect the influence of real-world variables on behavior, thus questioning the external validity of our results.

When interpreting the results, it is crucial to consider that although both groups were highly trained, the number of completed sessions varied, with the prepared group completing up to 17 sessions (almost 10,000 trials). Despite meeting the predetermined sample size requirements, the statistical power of the sample should be interpreted with caution. The replicability of the findings, particularly concerning the between-subject effects observed between the two groups, remains to be determined through further research.

Furthermore, while the study observed a high error rate with triple tasks, this does not necessarily undermine the results' legitimacy or the outcomes' quality. High error rates can indicate the complexity and challenge of triple tasks, which require participants to juggle multiple tasks simultaneously. Despite the high error rates, the study yielded significant findings and demonstrated the potential benefits of training on task performance. Therefore, while the high error rate is a limitation, it does not detract from the overall value and contributions of the study. In light of these considerations, our study should be seen as a stepping stone, offering preliminary insights while also highlighting the need for further, more extensive research to build upon and extend our understanding of multi-tasking across diverse and complex environments.

### Conclusion

Our investigation highlights the need to consider task complexity for future explorations in multi-tasking research. An increase in the number of task sets substantially affects RTs, particularly in participants who have not undergone task-specific training. Those with dual-task preparation initially exhibited superior performance, but this advantage diminished over time. Intriguingly, different strategies for managing the triple tasks emerged between the two groups, with more task-set combinations presenting a notable challenge to coordination efforts. The results suggest that individuals with skills such as task coordination acquired in specific subtasks can indeed benefit from scenarios with new tasks, given sufficient training. Moreover, the formation of task sets (or maybe task set pairs) seems to play a pivotal role in processing and coordinating tasks. Once established and expanded (with a new task), it influences the establishment of further task sets (pairs). This resilience poses a considerable challenge for current cognitive models to accurately predict the interaction between stimulus–response sets and their corresponding RTs. Our research underscores the necessity for additional studies that delve into the dynamics of inter(sub)task coordination and the prioritization of multiple tasks. As multi-tasking remains an essential aspect of daily life, the continuous refinement of cognitive models is paramount to keep pace with discoveries and the ever-growing complexity of multi-tasking behaviors. Moving forward, the adaptability of these models will be critical in understanding and predicting human behavior in an increasingly multitasking-oriented world.

## Data Availability

The datasets, material, and code generated as part of our study and/or analyzed during the current study are available via the Open Science Framework at: https://osf.io/fr4wa/. This study was not pre-registered.

## Appendix 1

**Table 2:** Repeated-measures ANOVA for response times of the visual-manual and auditory-vocal task in the preparation phase with Greenhouse–Geisser corrections for violations of sphericity with the within-subject factors complexity (simple and medium) and session (1–9)

		df		F	p	η^2^p
**Visual-manual task**						
Session	2.33, 28.00			6.52	0.003	0.352
Complexity	1, 12			0.84	0.378	0.065
Session ✻ Complexity	1.95, 23.38			10.12	< 0.001	0.457
**Auditory-vocal task**						
Session	2.48, 29.81			4.92	0.01	0.291
Complexity	1, 12			18.98	< 0.001	0.613
Session ✻ Complexity	2.89, 34.62			2.28	0.098	0.16

**Table 3:** Repeated-measures ANOVA for error rates of the visual-manual and auditory-vocal task in the preparation phase with Greenhouse–Geisser corrections for violations of sphericity with the within-subject factors complexity (simple and medium) and session (1–9)

	df		F	p	η^2^p
**Visual-manual task**					
Session	1.86, 22.28		5.64	0.012	0.32
Complexity	1, 12		0.31	0.589	0.025
Session ✻ Complexity	3.13, 37.56		1.58	0.208	0.117
**Auditory-vocal task**					
Session	1.48, 17.78		0.47	0.578	0.037
Complexity	1, 12		1.38	0.262	0.103
Session ✻ Complexity	3.05, 36.57		1.44	0.247	0.107

**Table 4:** Repeated-measures ANOVA for response times of the visual-manual, visual-pedal, and auditory-vocal task in the assessment phase with Greenhouse–Geisser corrections for violations of sphericity with the within-subject factors complexity (simple and medium) and session (1–8) and the between-subject factor preparation status (unprepared and prepared)

		df		F	p	η^2^p
**Visual-manual task**						
Session	2.96, 73.95			99.42	< 0.001	0.799
Session ✻ Preparation status	2.96, 73.95			5.90	0.001	0.191
Complexity	1.02, 25.48			36.31	< 0.001	0.592
Complexity ✻ Preparation status	1.02, 25.48			0.62	0.441	0.024
Session ✻ Complexity	3.43, 85.76			32.56	< 0.001	0.566
Session ✻ Complexity ✻ Preparation status	3.43, 85.76			1.23	0.306	0.047
Preparation status	1.00, 25			1.99	0.171	0.074
**Visual-pedal task**						
Session	2.51, 62.75			55.17	< 0.001	0.688
Session ✻ Preparation status	2.51, 62.75			5.39	0.004	0.177
Complexity	1.14, 28.39			165.35	< 0.001	0.869
Complexity ✻ Preparation status	1.14, 28.39			7.80	0.007	0.238
Session ✻ Complexity	5.28, 132.06			7.55	< 0.001	0.232
Session ✻ Complexity ✻ Preparation status	5.28, 132.06			2.51	0.030	0.091
Preparation status	1.00, 25			2.14	0.156	0.079
**Auditory-vocal task**						
Session	3.29, 82.15			37.88	< 0.001	0.602
Session ✻ Preparation status	3.29, 82.15			2.70	0.046	0.097
Complexity	1.12, 27.88			83.75	< 0.001	0.77
Complexity ✻ Preparation status	1.12, 27.88			5.70	0.021	0.186
Session ✻ Complexity	5.04, 125.95			13.27	< 0.001	0.347
Session ✻ Complexity ✻ Preparation status	5.04, 125.95			1.28	0.276	0.049
Preparation status	1.00, 25			11.76	0.002	0.32

**Table 5:** Repeated-measures ANOVA for error rates of the visual-manual, visual-pedal, and auditory-vocal task in the assessment phase with Greenhouse–Geisser corrections for violations of sphericity with the within-subject factors complexity (simple and medium) and session (1–8) and the between-subject factor preparation status (unprepared and prepared)

	df		F	p	η^2^p
**Visual-manual task**					
Session	3.40, 84.93		8.47	< 0.001	0.253
Session ✻ Preparation status	3.40, 84.93		0.33	0.83	0.013
Complexity	1.29, 32.20		4.92	0.026	0.164
Complexity ✻ Preparation status	1.29, 32.20		2.63	0.107	0.095
Session ✻ Complexity	7.07, 176.70		0.46	0.865	0.018
Session ✻ Complexity ✻ Preparation status	7.07, 176.70		0.90	0.511	0.035
Preparation status	1.00, 25		0.00	0.971	0
**Visual-pedal task**					
Session	4.68, 117.10		2.54	0.035	0.092
Session ✻ Preparation status	4.68, 117.10		1.97	0.093	0.073
Complexity	1.77, 44.12		33.74	< 0.001	0.574
Complexity ✻ Preparation status	1.77, 44.12		4.19	0.026	0.143
Session ✻ Complexity	7.96, 198.93		3.79	< 0.001	0.132
Session ✻ Complexity ✻ Preparation status	7.96, 198.93		0.93	0.494	0.036
Preparation status	1.00, 25		2.52	0.125	0.092
**Auditory-vocal task**					
Session	3.01, 75.26		1.60	0.196	0.06
Session ✻ Preparation status	3.01, 75.26		0.49	0.693	0.019
Complexity	1.68, 41.92		9.42	< 0.001	0.274
Complexity ✻ Preparation status	1.68, 41.92		1.01	0.362	0.039
Session ✻ Complexity	6.58, 164.41		4.15	< 0.001	0.142
Session ✻ Complexity ✻ Preparation status	6.58, 164.41		0.92	0.489	0.035
Preparation status	1.00, 25		0.12	0.732	0.005

## Footnotes

1 Participants were informed at the beginning that they would be taking part in two phases running over two semesters. However, some exercised their right not to continue participating and did not participate in the assessment phase in the second semester. No exclusion was made by the test administration.
